# FIGO/ICM guidelines for preventing Rhesus disease: A call to action

**DOI:** 10.1002/ijgo.13459

**Published:** 2021-01-09

**Authors:** Gerard H. A. Visser, Trude Thommesen, Gian Carlo Di Renzo, Anwar H. Nassar, Steven L. Spitalnik, FIGO Committee for Safe Motherhood, Newborn Health, Anwar Nassar, Gerard H. A Visser, Eytan Barnea, Maria Fernanda Escobar, Yoon Ha Kim, Wanda Kay Nicholson, R. Pacagnella, Diana Ramasaukaite, Gerhard Theron, Alison Wright

**Affiliations:** ^1^ International Federation of Gynecology and Obstetrics London UK; ^2^ Worldwide Initiative for Rh Disease Eradication New York NY USA; ^3^ International Confederation of Midwives The Hague the Netherlands

**Keywords:** Anti‐D immunoglobulin, FIGO, Guidelines, International Confederation of Midwives, Prophylaxis, Rhesus disease, Worldwide Initiative for Rhesus Disease Eradication

## Abstract

The introduction of anti‐Rh(D) immunoglobulin more than 50 years ago has resulted in only a 50% decrease in Rhesus disease globally owing to a low uptake of this prophylactic approach. The International Federation of Gynecology and Obstetrics, International Confederation of Midwives, and Worldwide Initiative for Rhesus Disease Eradication have reviewed current evidence regarding the utility of anti‐Rh(D) immunoglobulin. Taking into account the effectiveness anti‐Rh(D), the new guidelines propose adjusting the dose for different indications and prioritizing its administration by indication.

## Abstract 2

These FIGO/ICM guidelines review the evidence regarding the usefulness of anti‐Rh(D) immunoglobulin, prioritizing its administration by indication.

## 1 INTRODUCTION

In 1968, more 50 years ago, anti‐Rh(D) immunoglobulin was approved for use among Rhesus (Rh)‐negative women to prevent sensitization to the Rh(D) blood group antigen after delivery.^1^, ^2^ Subsequently, this approach was expanded to give anti‐Rh(D) prophylaxis during pregnancy to prevent sensitization in the third trimester, as well as anti‐D prophylaxis in the case of miscarriage, ectopic pregnancy, amniocentesis, bleeding or abdominal trauma during pregnancy, and/or external cephalic version for breech presentation. Recently in some countries, fetal Rh determination in maternal blood has been introduced in early pregnancy to prevent unnecessary immunoglobulin administration when the fetus seems to be Rh(D)‐negative.^3^

This approach is highly effective and Rh disease has been more or less eradicated in most high‐income countries. Nevertheless, recent data have shown that, in approximately 50% of eligible cases worldwide, anti‐Rh(D) immunoglobulin is not administered.^4^, ^5^ The reasons vary but include insufficient supply, cost considerations, ignorance (e.g., simply forgot to administer anti‐Rh[D]), lack of access, and use of products that have not been tested for therapeutic efficacy.^6^ It has been estimated that Rh disease still results in more than 160 000 perinatal deaths and 100 000 cases of disability annually, representing only a 50% reduction relative to the era before immunoglobulin administration.^4^ Such a high burden of a preventable disease should be considered completely unacceptable.

The aim of the present study was to summarize data on the prevention of Rh disease by immunoprophylaxis and provide guidelines that take into consideration the cost‐effectiveness of the different dose regimens and prioritize the administration of anti‐Rh(D) by indication. The guidelines are summarized in Box 1.

Box 1 **Measures to prevent sensitization to Rh(D)**

**High priority**

Determine the maternal Rh factor, preferably in early pregnancy.

For Rh(D)‐negative women, determine the Rh factor of the newborn from umbilical cord blood.

Administer anti‐Rh(D) immunoglobulin within 72 hours of delivery to women with a Rh(D)‐positive newborn, unless already sensitized.

Use a dose of 500 IU (100 µg) of anti‐Rh(D) immunoglobulin; if affordable and with sufficient supply, 1500 IU (300 µg) may be given, as is common in high‐income countries. The intramuscular route is as effective as the intravenous route.

**Middle priority**

Routine anti‐Rh(D) prophylaxis during pregnancy: 1500 IU (300 µg) at 28–34 weeks.

Anti‐Rh(D) immunoglobulin prophylaxis (500 IU; 100 µg) after a surgical abortion or ectopic pregnancy (all gestational ages), or after spontaneous or medical abortion/miscarriage after 10 weeks.

Anti‐Rh(D) prophylaxis after bleeding, abdominal trauma in pregnancy, and/or fetal death (500 or 1500 IU; 100 or 300 µg) during the second or third trimester. Kleihauer–Betke test can be used to estimate the optimal dose.

**Low priority**

Anti‐Rh(D) prophylaxis after amniocentesis, chorionic villus sampling, or external cephalic version (500 IU; 100 µg).

## 2 BLOOD GROUP AND RH(D) TYPING

A pre‐requisite for the prevention of Rh(D) sensitization is a priori knowledge of maternal Rh status. Although this is widely agreed upon, it is not the case in many low‐resource settings. The Rh(D) factor can be determined by collecting venous or capillary blood samples at local healthcare facilities and using classical or point‐of‐care serologic methods. The Rh(D) type should preferably be determined in the first trimester, because indications for anti‐Rh(D) immunoprophylaxis may arise early in pregnancy; for example, after a miscarriage or an ectopic pregnancy.

## 3 POSTPARTUM ANTI‐RH(D) IMMUNOGLOBULIN ADMINISTRATION

Rh(D) sensitization occurs in approximately 16% of pregnancies among Rh(D)‐negative women. Postpartum administration of anti‐Rh(D) immunoglobulin reduces this risk to approximately 1.5%,^3^ and is the most effective intervention to prevent Rh disease in subsequent pregnancies. Therefore, this approach should have the highest priority in countries and/or regions where no, or inadequate, prophylaxis is currently provided. When an Rh(D)‐positive neonate is delivered by an Rh(D)‐negative woman, 1500 IU (equivalent to 300 µg) of anti‐Rh(D) should be administered intramuscularly within 72 hours after delivery. This is sufficient to neutralize 30 mL of Rh(D)‐positive fetal whole blood.^7^ According to one study, the median fetal–maternal transfusion at delivery is approximately 0.7 mL, with a transfusion exceeding 10 mL in only approximately 1% of cases.^8^ Therefore, it has been suggested that an anti‐Rh(D) dose of 500 IU (100 µg) would be sufficient. Nonetheless, the effectiveness of administering a higher standard dose has not been established,^9^, ^10^ although a recent meta‐analysis suggests that the 1500 IU regimen has slightly better efficacy.^11^

In some countries, it is policy to give a double dose of anti‐Rh(D) after a cesarean delivery. However, this does not seem to be necessary because data from a large study in the Czech Republic did not show a greater volume of fetal–maternal transfusion after cesarean delivery.^8^ In case of uncertainty, a Kleihauer–Betke test may be performed to estimate the actual volume of fetal–maternal transfusion. It has been calculated that one vial of 1500 IU will prevent sensitization by 30 mL of fetal whole blood. This test is also reasonable in other settings where there is uncertainty regarding the size of a fetal–maternal hemorrhage (e.g., intrauterine fetal death). In the Kleihauer–Betke test, the percentage of fetal cells in maternal circulation is calculated by counting the number of fetal red cells in a maternal blood smear as follows: % of fetal red cells relative to maternal red cells × 50 = amount of fetal whole blood in maternal circulation (in mL).

## 4 ANTI‐RH(D) IMMUNOGLOBULIN ADMINISTRATION IN PREGNANCY

Most cases of Rh(D) sensitization occur as a result of labor. Routine prenatal administration of anti‐Rh(D) immunoglobulin to prevent sensitization resulting from fetal–maternal hemorrhage during pregnancy has been studied in a meta‐analysis of two randomized controlled trials.^12^ These showed a 42% reduction in sensitization, although this reduction was not significant (95% confidence interval [CI], 0.15–1.62).^12^ However, a ‘bias‐adjusted’ meta‐analysis of data from 10 studies estimated a pooled odds ratio for a reduction in sensitization of 0.31 (95% CI, 0.17–0.56), which was highly significant.^13^ Therefore, prenatal administration seems to reduce sensitization further, from approximately 1.5%, achieved by administration of postpartum anti‐Rh(D) immunoglobulin, to approximately 0.5%.

Prenatal anti‐Rh(D) immunoglobulin may be given intramuscularly or intravenously, with no clear difference in effectiveness.^14^ It may be given once at 28–34 weeks of gestation (1500 IU), or twice at 28 and 32–34 weeks (625 IU or 1500 IU at each gestational age). Two recent meta‐analyses and an additional randomized controlled trial showed that a single administration of 1500 IU resulted in the lowest proportion of women with detectable circulating anti‐Rh(D) at delivery, suggesting that this is the optimal dose against sensitization during pregnancy.^11^, ^13^, ^15^

## 5 MISCARRIAGE

The risk for sensitization is most probably extremely low for spontaneous abortions before 10 gestational weeks^16^; however, data are scarce. Based on the clinical expertise of the guideline committee from the UK’s National Institute for Health and Care Excellence (NICE), it is suggested that prophylaxis should be given only to women who are having a spontaneous abortion or medical management of miscarriage after 10^0/7^ gestational weeks. Moreover, for women who have surgical management, prophylaxis may also be considered before 10 gestational weeks.^16^ Given the low fetal blood volume during early gestation, an anti‐Rh(D) immunoglobulin dose of 500 IU may be used, although there are no data to support this policy.

In a complete molar pregnancy, organogenesis does not occur; thus, sensitization to Rh(D) should not occur. However, the situation is different in a partial molar pregnancy. Because differentiating between the forms of molar pregnancy may be difficult, it is generally advised to administer anti‐Rh(D) immunoglobulin in this setting.^3^

## 6 ECTOPIC PREGNANCY

A ruptured tubal pregnancy has been associated with a 24% incidence of alloimmunization to Rh(D) among Rh(D)‐negative women.^17^ Therefore, anti‐Rh(D) immunoglobulin administration is strictly advised for ectopic pregnancy. Because fetal blood volume is low in early gestation, the dose of anti‐Rh(D) required may be low.

## 7 CHORIONIC VILLUS SAMPLING OR AMNIOCENTESIS

Most countries advocate administering anti‐Rh(D) immunoglobulin to Rh(D)‐negative pregnant women after chorionic villus sampling or amniocentesis, although this recommendation is based on limited scientific evidence.^3^ In Denmark, by contrast, immunoprophylaxis is not provided in this setting because no differences in alloimmunization at 29 weeks were found between women with invasive testing and those without (900 cases would be needed to prevent one case of immunization).^18^

## 8 BLEEDING AND ABDOMINAL TRAUMA IN PREGNANCY

Abdominal trauma may cause fetal–maternal transfusion, which might lead to Rh(D) alloimmunization. Although the exact risks are unknown, it is advised to administer anti‐Rh(D) immunoglobulin as prophylaxis. The same holds for prenatal hemorrhage in the second and third trimester.^3^ The optimal dose of anti‐Rh(D) immunoglobulin is not known (1500 IU is most commonly used).

## 9 INTRAUTERINE FETAL DEATH

Because an intrauterine fetal death may have been caused by a large fetal–maternal hemorrhage, it may be useful to perform a Kleihauer–Betke test, both as a part of the workup of the fetal death and — among Rh(D)‐negative women — to determine the amount of fetal–maternal hemorrhage to calculate the dose of anti‐Rh(D) immunoglobulin needed.

## 10 EXTERNAL CEPHALIC VERSION IN BREECH PRESENTATION

The risk of fetal–maternal transfusion during external cephalic version ranges from 2% to 6%^19^, ^20^; therefore, administration of anti‐Rh(D) immunoglobulin is advised.^3^ However, the amount of transfusion is generally low. Based on a large Canadian study,^19^ it has been concluded that routine administration of prenatal anti‐Rh(D) immunoglobulin at approximately 32 gestational weeks should be enough to prevent sensitization during a subsequent external cephalic version.

## 11 NONINVASIVE FETAL RH(D) TYPING IN THE FIRST TRIMESTER

Non‐invasive prenatal testing of cell‐free DNA in the first trimester of pregnancy may be used to determine fetal Rh(D) status. Such a policy has recently been introduced into clinical practice in countries such as Denmark, the Netherlands, and the United Kingdom. A recent meta‐analysis of 60 000 participants showed that it has a very high sensitivity (99.9%; 95% CI, 99.5%–100%) and specificity (99.2%; 95% CI, 89.5%–99.5%) as compared with testing newborn's blood.^21^ First‐trimester non‐invasive Rh(D) typing may therefore be used to prevent unnecessary administration of anti‐Rh(D) immunoglobulin in the course of pregnancy (routinely or following amniocentesis, etc.). Although population‐based cell‐free DNA as a method to determine Rh status may not be currently cost‐effective in all settings,^3^ health policymakers should include this non‐invasive test as a future option for combating Rh disease.

## 12 DOSAGE OF ANTI‐RH(D) IMMUNOGLOBULIN

Surprisingly little is known about the optimal dose of anti‐Rh(D) immunoglobulin. Postpartum, a dose of 1500 IU may be slightly better than 500 IU, but financial restriction may prompt use of the lower dose. In early pregnancy, the amount of fetal–maternal hemorrhage is bound to be low; therefore, a dose of 500 IU should generally be enough. Prophylaxis in the third trimester should optimally consist of a dose of 1500 IU given once between 28 and 34 weeks. No information is available on the immunoglobulin dose that should be given after maternal vaginal bleeding, abdominal trauma, or fetal death. However, the Kleihauer–Betke test is very useful and provides dosing guidance for abdominal trauma or fetal death.

## 13 MEASURES TO PREVENT ANTI‐RH(D) SENSITIZATION

Box 1 summarizes the measures to prevent anti‐Rh(D) sensitization, taking into account the cost‐effectiveness of the different dose regimens and prioritizing the administration of anti‐Rh(D) by indication. When studying the gap between the annual doses of anti‐Rh(D) given and the annual doses required, it can be concluded that the highest priority is met only in high‐income countries and countries such as Brazil, Czech Republic, Croatia, Greece, Hungary, Iran, Lithuania, Malaysia, Saudi Arabia, Sri Lanka, South Korea, Thailand, Turkey, and Uruguay.^5^ There is still a long way to go.

## CONFLICTS OF INTEREST

The authors have no conflicts of interest.

## AUTHOR CONTRIBUTIONS

GHAV wrote the manuscript, which was modified/amended by TT, GC DR, AN, and SLS and approved by the FIGO Committee of Safe Motherhood and Newborn Health.

## COMMITTEE MEMBERS

Anwar Nassar (Chair), Gerard H. A. Visser (Past chair), Eytan Barnea, Maria Fernanda Escobar, Yoon Ha Kim, Wanda Kay Nicholson, R. Pacagnella, Diana Ramasaukaite, Gerhard Theron, Alison Wright.
